# Oral administration of Robinia pseudoacacia L. flower exosome-like nanoparticles attenuates gastric and small intestinal mucosal ferroptosis caused by hypoxia through inhibiting HIF-1α- and HIF-2α-mediated lipid peroxidation

**DOI:** 10.1186/s12951-024-02663-6

**Published:** 2024-08-12

**Authors:** Dezhi Wang, Heng Zhang, Xingchen Liao, Jun Li, Jie Zeng, Yilin Wang, Mingjie Zhang, Xianzong Ma, Xin Wang, Fangli Ren, Yinyin Wang, Meng Li, Junfeng Xu, Peng Jin, Jianqiu Sheng

**Affiliations:** 1 Medical School of Chinese PLA, Chinese PLA General Hospital, Road Fuxing No. 28, Haidian District, Beijing, 100853 China; 2 Department of Gastroenterology, The Seventh Medical Center of Chinese PLA General Hospital, Beijing, 100700 China; 3 State Key Laboratory of Membrane Biology, School of Medicine, Institute of Precision Medicine, Tsinghua University, Beijing, 100084 China; 4 Senior Department of Gastroenterology, The First Medical Center of Chinese PLA General Hospital, Road Fuxing No. 28, Haidian District, Beijing, 100853 China; 5 Department of Urology, The Second Affiliated Hospital, School of Medicine, South China University of Technology, Guangzhou, 510180 China

**Keywords:** Robinia pseudoacacia L. flower exosome-like nanoparticles, Hypoxia, Hypoxia-inducible factor (HIF), Arachidonate lipoxygenase 5 (ALOX5), Nicotinamide adenine dinucleotide phosphate oxidase 4 (NOX4), Stomach, Small intestine, Ferroptosis

## Abstract

The prevention and treatment of gastrointestinal mucosal injury caused by a plateau hypoxic environment is a clinical conundrum due to the unclear mechanism of this syndrome; however, oxidative stress and microbiota dysbiosis may be involved. The Robinia pseudoacacia L. flower, homologous to a functional food, exhibits various pharmacological effects, such as antioxidant, antibacterial, and hemostatic activities. An increasing number of studies have revealed that plant exosome-like nanoparticles (PELNs) can improve the intestinal microbiota and exert antioxidant effects. In this study, the oral administration of Robinia pseudoacacia L. flower exosome-like nanoparticles (RFELNs) significantly ameliorated hypoxia-induced gastric and small intestinal mucosal injury in mice by downregulating hypoxia-inducible factor-1α (HIF-1α) and HIF-2α expression and inhibiting hypoxia-mediated ferroptosis. In addition, oral RFELNs partially improved hypoxia-induced microbial and metabolic disorders of the stomach and small intestine. Notably, RFELNs displayed specific targeting to the gastrointestinal tract. In vitro experiments using gastric and small intestinal epithelial cell lines showed that cell death caused by elevated HIF-1α and HIF-2α under 1% O_2_ mainly occurred via ferroptosis. RFELNs obviously inhibited HIF-1α and HIF-2α expression and downregulated the expression of NOX4 and ALOX5, which drive reactive oxygen species production and lipid peroxidation, respectively, suppressing ferroptosis under hypoxia. In conclusion, our findings underscore the potential of oral RFELNs as novel, naturally derived agents targeting the gastrointestinal tract, providing a promising therapeutic approach for hypoxia-induced gastric and small intestinal mucosal ferroptosis.

**Graphical Abstract:**

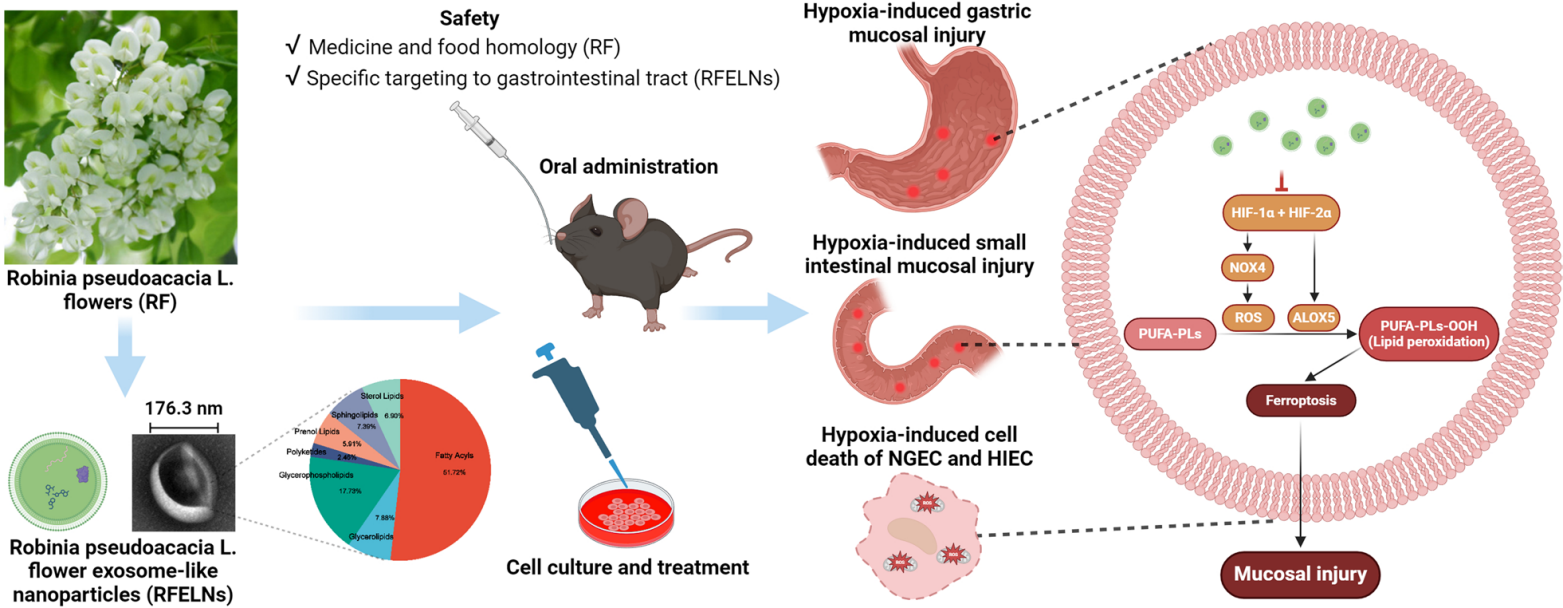

**Supplementary Information:**

The online version contains supplementary material available at 10.1186/s12951-024-02663-6.

## Introduction

High-altitude environments can cause gastrointestinal disorders, such as anorexia, nausea, vomiting, epigastric discomfort, dyspepsia, diarrhea, and fecal occult blood [1–3], mainly due to hypoxia and hypoxia-induced reductive oxidative-mediated gastrointestinal mucosal barrier injury [4]. However, gastrointestinal mucosal barrier injury caused by a plateau hypoxic environment is difficult to prevent and control due to the unclear underlying mechanism. The maintenance of cellular homeostasis under hypoxia is mainly dependent on the regulation of hypoxia-inducible factors (HIFs), which consist of HIF-α subunits (HIF-1α and HIF-2α) and HIF-1β heterodimers. HIFs regulate the transcription of genes under hypoxic conditions by binding to hypoxia response elements on the promoters of specific genes [5]. Acute hypoxic conditions can increase HIF-1α and HIF-2α expression to enhance cellular adaptation to hypoxic environments [6]. However, if hypoxia persists, high expression of HIF-1α and HIF-2α leads to cell death [7]. Ferroptosis is programmed cell death characterized by iron-dependent lipid peroxidation and is executed by peroxidized membrane phospholipids (PLs) containing polyunsaturated fatty acyl (PUFA) chains. Reactive oxygen species (ROS) can stabilize the expression of HIFs by promoting the oxidative inactivation of prolyl hydroxylases (PHDs) [8]. In turn, HIF-1α and HIF-2α can increase ROS levels [9–12]. Elevated ROS levels could modulate PUFAs/PLs peroxidation, thereby inducing ferroptosis [13]. However, HIF-1α and HIF-2α have been reported to inhibit ferroptosis in tumor and normal cells [14–18]. The opposite effects of HIF-1α and HIF-2α on ferroptosis are potentially cell type–dependent [19]. The nicotinamide adenine dinucleotide phosphate (NADPH) oxidase (NOX) protein family produces ROS that participate in the regulation of intracellular redox balance and ferroptosis [20, 21]. The arachidonate lipoxygenase (ALOX) family and cytochrome P450 oxidoreductase (POR, also known as CYPOR) mediate ferroptosis through the peroxidation of PUFAs [22].

Robinia pseudoacacia L. flowers are commonly used as a traditional medicine, homologous to functional food [23–25]. Robinia pseudoacacia L. flowers contain multiple pharmacologic flavonoid components, such as rutin, hyperoside, and quercetin [26], and exhibit a wide range of pharmacological effects, such as antioxidant, antibacterial, and hemostatic activities [27, 28]. Plant exosome-like nanoparticles (PELNs), released by numerous plants, can exert immunomodulatory effects, improve the intestinal microbiota, exert regenerative effects on interspecies communication, and demonstrate antioxidant effects through their biomolecule content (lipids, proteins, and microRNAs) [29–32]. PELNs offer several advantages over other approaches, such as low toxicity, reduced immunogenicity, efficient cellular uptake, and high biocompatibility and stability [29, 33–36]. These properties make PELNs highly promising for clinical applications in the treatment of gastrointestinal diseases. For example, grape exosome-like nanoparticles have been shown to induce the recovery of intestinal stem cells through the Wnt/β-catenin signaling pathway and protect mice from DSS-induced colitis [37]. Plant-derived exosomal microRNAs can shape the gut microbiota, which is linked to improvements in barrier function [38]. Considering that Robinia pseudoacacia L. flowers exhibit pharmacological effects, including antioxidation effects and the restoration of gut microbiota dysbiosis [27, 28], we hypothesized that Robinia pseudoacacia L. flower exosome-like nanoparticles (RFELNs) have considerable potential in the treatment of hypoxia-induced gastrointestinal mucosal barrier dysfunction. Therefore, our study aimed to explore the effects and mechanisms of RFELNs in hypoxia-induced gastrointestinal mucosal cellular barrier injury in mouse and cell models.

## Methods

### Isolation and identification of RFELNs

Fresh Robinia pseudoacacia L. flowers were purchased from Beijing, China. RFELNs were isolated via ultracentrifugation. Briefly, Robinia pseudoacacia L. flowers were cut, washed, and allowed to dry under sterile conditions. Then, the samples were chopped and immersed in 75% alcohol for 1 min, after which they were washed with distilled water to remove the alcohol residue, dried with sterile paper, supplemented with 20 mL of enzyme solution (4% cellulase, 2% pectinase, and 0.6 mol/L mannitol, pH 5.8), and enzymatically digested at 50 ℃ for 6 h. The samples were subsequently centrifuged (4 ℃, 2000 × *g*, 30 min), and the supernatant was carefully transferred to a new tube and centrifuged again (4 ℃, 10,000 × *g*, 45 min) to remove larger vesicles and dead cells from the supernatant. Subsequently, the supernatant was filtered through a 0.45 μm membrane and ultracentrifuged (4 ℃, 100,000 × *g*, 70 min) twice. The sediment was resuspended in 150 μL of precooled 1 × phosphate-buffered saline (PBS); 20 μL of RFELNs was removed for observation via transmission electron microscopy (TEM), 10 μL for particle size measurement, and the remainder was stored at – 80 ℃. Untargeted metabolomics analysis was performed using a high-performance liquid chromatograph (System: Thermo Scientific^™^ Dionex^™^ ΜltiMate^™^ 3000 rapid separation liquid chromatography, Thermo Fisher Scientific, Waltham, MA, USA) and mass spectrometry system (Model: Q Exactive, Thermo Fisher Scientific, Waltham, MA, USA) in conjunction with the Human Metabolome Database (HMDB, http://www.hmdb.ca/) and LIPID MAPS Structure Database (LMSD, http://www.lipidmaps.org/data/structure/) to clarify RFELNs metabolite fractions.

### Mice

C57BL/6 J mice (male and female, 6 weeks old) were obtained from Charles River Laboratories, Beijing, China, and housed in a specific-pathogen-free environment with a 12-h light/dark cycle and free access to food and water. Mice were housed for 1 week to adapt to the conditions before the experiments. The experimental protocols were approved by the Animal Care and Use Committee of Tsinghua University (Protocol # 23-CZJ1).

### Establishment of the hypoxic animal model

Hypoxia-induced gastric and small intestinal injury were triggered by placing the mice in a ventilated hypoxic chamber system (LP-1500, Yuyan Instruments, Shanghai, China) to simulate an altitude of 5500 m above sea level for 3 days. Mice were randomly divided into five groups: the normoxia group, which was exposed to room air (21% O_2_) for 3 days and gavaged with 100 μL of PBS on day 1; the hypoxia group, which was placed in a hypoxic chamber for 3 days and gavaged with 100 μL of PBS on day 1; the low-dose group, which was placed in a hypoxic chamber for 3 days and gavaged with 5 × 10^4^ particles of RFELNs in 100 μL of PBS on day 1; the medium-dose group, which was placed in a hypoxic chamber for 3 days and gavaged with 1 × 10^5^ particles of RFELNs in 100 μL of PBS on day 1; and the high-dose group, which was placed in a hypoxic chamber for 3 days and gavaged with 5 × 10^5^ particles of RFELNs in 100 μL of PBS on day 1. Food consumption, body weight, stool score, and stool blood score data were recorded daily. Mice were euthanized on day 3, and the entire gastrointestinal tract, including the esophagus, stomach, duodenum, jejunum, ileum, and colon, was removed for hematoxylin and eosin (H&E) staining. Gastric and small intestinal tissues were harvested for TEM. Gastric and small intestinal mucosa samples were collected for western blot, multiplex ELISA, and peroxide detection. Gastric and small intestinal contents were collected for 16S rRNA sequencing, metabolomics analysis, and combined microbial and metabolomic analysis. The disease activity index (DAI) and histological index were scored as previously described [39, 40].

### Cell culture and treatments

NGEC (human normal gastric epithelial cell line) and HIEC (human normal small intestinal epithelial cell line) were purchased from Otwo Biotech, Shenzhen, Guangdong, China, and cultured in DMEM supplemented with 10% fetal bovine serum, 100 U/mL penicillin G, and 100 μg/mL streptomycin sulfate (all from Corning, New York, NY, USA). The cells were cultured in a humidified atmosphere at 37 ℃ with 21% O_2_ and 5% CO_2_ under normoxic conditions. Hypoxia-treated cells were incubated with 1% O_2_ and 5% CO_2_. After 24 h of hypoxia, the cells were subjected to different concentrations of RFELNs (5 × 10^3^ particles/mL, 2.5 × 10^4^ particles/mL, and 5 × 10^4^ particles/mL) or PBS and analyzed using crystal violet staining, cell counting kit-8 (CCK-8), western blot, peroxide detection, TEM, and lipidomic analysis after exposure to hypoxia for another 24 h. For the transfection experiments, NGEC and HIEC were transiently transfected with HIF-1α and HIF-2α using the pCMV vector plasmid, pCMV-HIF-1α plasmid (containing full-length wild-type cDNA of hHIF-1α), pCMV-HIF-2α plasmid (containing full-length wild-type cDNA of hHIF-2α), siNC, siRNA targeting HIF-1α, or siRNA targeting HIF-2α (siRNA sequence information is provided in Table S1). 24 h after transfection under normoxia, the cells were incubated under hypoxia for 24 h and treated with dimethyl sulfoxide (DMSO), ferrostatin-1 (Fer-1, 5 μM, S7243, Selleck, Beijing, China), or liproxstatin-1 (Lip-1, 1 μM, S7699, Selleck) under hypoxia for another 24 h. The cells were harvested for crystal violet staining and CCK-8 assay analysis.

### Gastrointestinal permeability in vivo

Mice were fasted for 4 h prior to gavage. FITC-dextran (MW 70000; MedChemExpress, Saint Louis, MO, USA) was administered by oral gavage at a concentration of 0.6 mg/*g* body weight. Serum was collected 4 h later, and fluorescence intensity was determined by fluorometry.

### 16S rRNA sequencing

The mice were euthanized, and the gastric and small intestinal contents were immediately isolated, frozen in liquid nitrogen, and stored at – 80 ℃ for subsequent DNA extraction and microbial analysis. Forward (5′-ACTCCTACGGGAGGCAGCA-3′) and reverse (5′-GGACTACHVGGGTWTCTAAT-3′) primers were used for amplification. The 16S rRNA gene sequences were analyzed using QIIME platform scripts (www.qiime.org). The reference sequences were selected into operational taxonomic units (OTUs) by clustering 97% sequence similarity. The ribosomal database project classifier was applied to systematically classify OTU sequences with reference to the Silva database.

### Analyses of metabolites of murine gastric and small intestinal contents

Liquid chromatography–mass spectrometry (LC‒MS) analysis was performed to identify the gastric and small intestinal contents of the mice. LC analysis was performed on a Vanquish UHPLC System (Thermo Fisher Scientific, Waltham, MA, USA). Chromatography was conducted using an ACQUITY UPLC ^®^ HSS T3 (2.1 × 100 mm, 1.8 µm) (Waters, Milford, MA, USA). The column was maintained at 40 ℃. The flow rate and injection volume were set at 0.3 mL/min and 2 μL, respectively. For LC-ESI ( +)–MS analysis, the mobile phases consisted of (B2) 0.1% formic acid in acetonitrile (v/v) and (A2) 0.1% formic acid in water (v/v). Separation was conducted under the following gradient: 0–1 min, 8% B2; 1–8 min, 8–98% B2; 8–10 min, 98% B2; 10–10.1 min, 98–8% B2; and 10.1–12 min, 8% B2. For LC-ESI (-)–MS analysis, the analytes were analyzed with (B3) acetonitrile and (A3) ammonium formate (5 mM). Separation was conducted under the following gradient: 0–1 min, 8% B3; 1–8 min, 8–98% B3; 8–10 min, 98% B3; 10–10.1 min, 98–8% B3; and 10.1–12 min, 8% B3 [41].

Mass spectrometric detection of metabolites was performed on an Orbitrap Exploris 120 (Thermo Fisher Scientific, USA) with an ESI ion source. Simultaneous MS1 and MS/MS (full MS-ddMS2 mode, data-dependent MS/MS) acquisition was used. The parameters were as follows: sheath gas pressure, 40 arb; aux gas flow, 10 arb; spray voltage, 3.50 kV and – 2.50 kV for ESI( +) and ESI(–), respectively; capillary temperature, 325 ℃; MS1 range, m/z 100–1000; MS1 resolving power, 60,000 FWHM; number of data-dependent scans per cycle, 4; MS/MS resolving power, 15,000 FWHM; normalized collision energy, 30%; and dynamic exclusion time, automatic [42].

### Combined analysis of microbial and metabolomic data

Differential microbiota analysis of murine gastric and small intestinal contents was conducted by assessing 16S rRNA, and differential metabolites were identified by LC‒MS. Correlation analyses of differential microbiota and metabolites were performed using R packages.

### Distribution of RFELNs in vivo* and *in vitro

DiR-labeled RFELNs were orally administered in 100 μL PBS at a single dose of 5 × 10^5^ particles/100 µL. The distribution of RFELNs was visualized by in vivo imaging using an Odyssey CLx imaging system (LI-COR Biosciences, Lincoln, NE, USA) at 0, 6, 16, and 24 h after administration.

The lipophilic dye PKH67 was used to label RFELNs in vitro. Adherent cells were treated with PKH67-labeled RFELNs (5 × 10^3^ particles/mL) and incubated in a cell culture incubator for 24 h. Then, the cells were fixed with 4% paraformaldehyde and observed under a confocal microscope (TCS SP8 CARS, Leica, Frankfurt, Hessian, Germany).

### Transmission electron microscopy

After the mice were euthanized, the gastric and small intestinal tissues were quickly removed, cut into 1 mm^3^ pieces, and fixed in 2.5% glutaraldehyde. NGEC and HIEC were fixed with 2.5% glutaraldehyde after the culture medium was removed. Tissues and cells were then osmium tetroxide-fixed, dehydrated in an alcohol gradient, permeabilized with acetone and epoxy resin, embedded in epoxy resin, cut into 80–100 nm ultrathin sections using an ultramicrotome (UC7, Leica), stained with 2% uranyl acetate saturated aqueous solution and lead citrate, and observed by TEM (TECNAI G 20 TWIN, FEI Company, Hillsboro, OR, USA).

### Western blot

Extracted protein (50 μg) from cells or mucosa was loaded onto polyacrylamide gels for electrophoresis and transferred to nitrocellulose membranes (Bio-Rad Laboratories, Hercules, CA, USA). Western blot was performed using primary antibodies targeting HIF-1α (1:1000, ab179483, Abcam, Carlsbad, MA, USA), HIF-2α (1:1000, PA1-16510, Invitrogen, Carlsbad, CA, USA), PHD1 (1:1000, ab113077, Abcam), PHD2 (1:1000, ab226890, Abcam), PHD3 (1:1000, ab184714, Abcam), GPX4 (1:1000, ab125066, Abcam), GAPDH (1:10000, ab8245, Abcam), NOX1 (1:1000, A11966, ABclonal, Wuhan, Hubei, China), NOX2 (1:1000, A19701, ABclonal), NOX4 (1:1000, A22149, ABclonal), ALOX5 (1:1000, A2877, ABclonal), ALOX12 (1:1000, A14703, ABclonal), ALOX15 (1:1000, A6864, ABclonal), CYPOR (1:1000, A5032, ABclonal), and a FITC-conjugated secondary antibody (1:10000 dilution; Millipore, Burlington, MA, USA). The blots were visualized using an ODYSSEY quantitative fluorescent imaging system (LI-COR Biosciences, Lincoln, NE, USA).

### Multiplex enzyme-linked immunosorbent assay (ELISA)

A multiplex ELISA kit for 32 cytokines/chemokines (MCYTMAG-70 K-PX32, Millipore, Burlington, MA, USA) was used to determine the levels of multiple cytokines/chemokines in the gastric and small intestinal mucosal extracts from mice.

### Detection of peroxides

ROS, 4-hydroxynonenal (4-HNE), and lipid hydroperoxide (LPO) levels in the gastric and small intestinal mucosa were analyzed according to the kit manufacturer’s instructions (ROS, E-BC-K138-F; 4-HNE, E-EL-0128c; LPO, E-BC-K176-M). ROS, 4-HNE, and malondialdehyde (MDA) levels in the NGEC and HIEC extracts were measured using a kit (ROS, E-BC-K138-F; 4-HNE, E-EL-0128c; MDA, E-BC-K028-M) according to the manufacturer’s instructions. All kits were purchased from Elabscience (Wuhan, Hubei, China).

### Crystal violet staining

Cells in 12-well plates were washed with PBS and fixed with 4% paraformaldehyde for 1 h, after which 1 mL of crystal violet solution (C0121, Beyotime Biotechnology, Shanghai, China) was added to each well for 30 min. The cells were washed three times with distilled water to remove excess crystal violet and observed under a scanner (V850P, Epson, Suwa, Nagano Prefecture, Japan).

### CCK-8 assay

NGEC and HIEC were seeded in 96-well plates, and cell viability was measured using a CCK-8 assay (Dojindo Laboratories, Shanghai, China) according to the manufacturer’s instructions.

### Lipidomic analysis

Lipids were extracted from approximately one million cells using a modified version of Bligh and Dyer’s method, as described previously [43]. Total protein content was determined from the dried pellet using the Pierce^®^ BCA Protein Assay Kit according to the manufacturer’s protocol. All lipidomic analyses were conducted by LipidALL Technologies using a Shimadzu Nexera 20AD-HPLC instrument coupled with a Sciex QTRAP 6500 PLUS, as reported previously [44]. Separation of individual lipid classes of polar lipids by normal phase (NP)-HPLC was conducted using a TUP-HB silica column (i.d. 150 × 2.1 mm, 3 µm) with the following conditions: mobile phase A (chloroform: methanol: ammonium hydroxide, 89.5:10:0.5) and mobile phase B (chloroform: methanol: ammonium hydroxide: water, 55:39:0.5:5.5). MRM transitions were set up for comparative analyses of various polar lipids. Individual lipid species were quantified by referencing spiked internal standards. Free fatty acids were quantitated using d31-16:0 (Sigma‒Aldrich, Darmstadt, Hessian, Germany) and d8-20:4 (Cayman Chemicals, Ann Arbor, MI, USA). Glycerol lipids, including diacylglycerols (DAGs) and triacylglycerols (TAGs), were quantified using a modified version of reversed-phase HPLC/MRM [45]. The levels of short-, medium-, and long-chain TAGs were calculated by referencing spiked internal standards of TAG(14:0)3-d5, TAG(16:0)3-d5, and TAG(18:0)3-d5 obtained from CDN isotopes, respectively. DAGs were quantified using d5-DAG17:0/17:0 and d5-DAG18:1/18:1 as internal standards (Avanti Polar Lipids, Alabaster, AL).

### Statistical analysis

Differences between the two groups were compared using Student’s *t*-test. The data from three or more groups were evaluated by one-way analysis of variance (ANOVA). Significance was considered at *P* < 0.05, and the data are expressed as the mean ± standard error of the mean (SEM).

## Results

### Isolation and identification of RFELNs

**Fig. 1:**
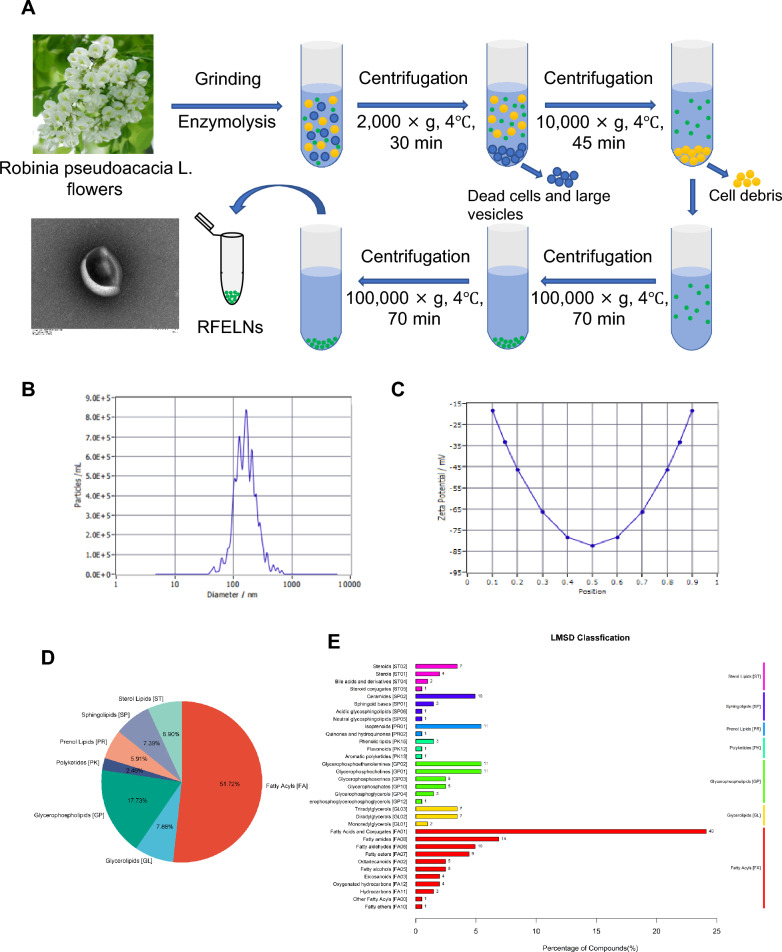
Isolation and identification of RFELNs. **A** Flowchart of RFELNs isolation by ultracentrifugation. **B** Analysis of particle size. **C** Analysis of surface charge. **D** Percentage of lipid components at the class level of RFELNs identified by liquid chromatography‒mass spectrometry (LC‒MS) combined with the LMSD database. **E** Specific composition of lipids at the subclass level of RFELNs

RFELNs were isolated via ultracentrifugation (Fig. 1A), and their morphology, particle size, and surface charge were determined via TEM and nanoparticle tracking analysis. The size of the RFELNs was 176.3 nm (Fig. 1B), and the zeta potential was – 83.4 mV (Fig. 1C). LC‒MS combined with the LMSD database was used to identify lipid components of RFELNs at the class and subclass levels, indicating that the RFELNs were rich in 51.72% fatty acyls, 17.73% glycerophospholipids, 7.88% glycerolipids, 7.39% sphingolipids, 6.90% sterol lipids, 5.91% prenol lipids, and 2.46% polyketides (Fig. 1D). The content of fatty acids and conjugates among fatty acyls reached 49% (Fig. 1E).

### RFELNs alleviated hypoxia-induced gastric and small intestinal mucosal injury in mice

**Fig. 2:**
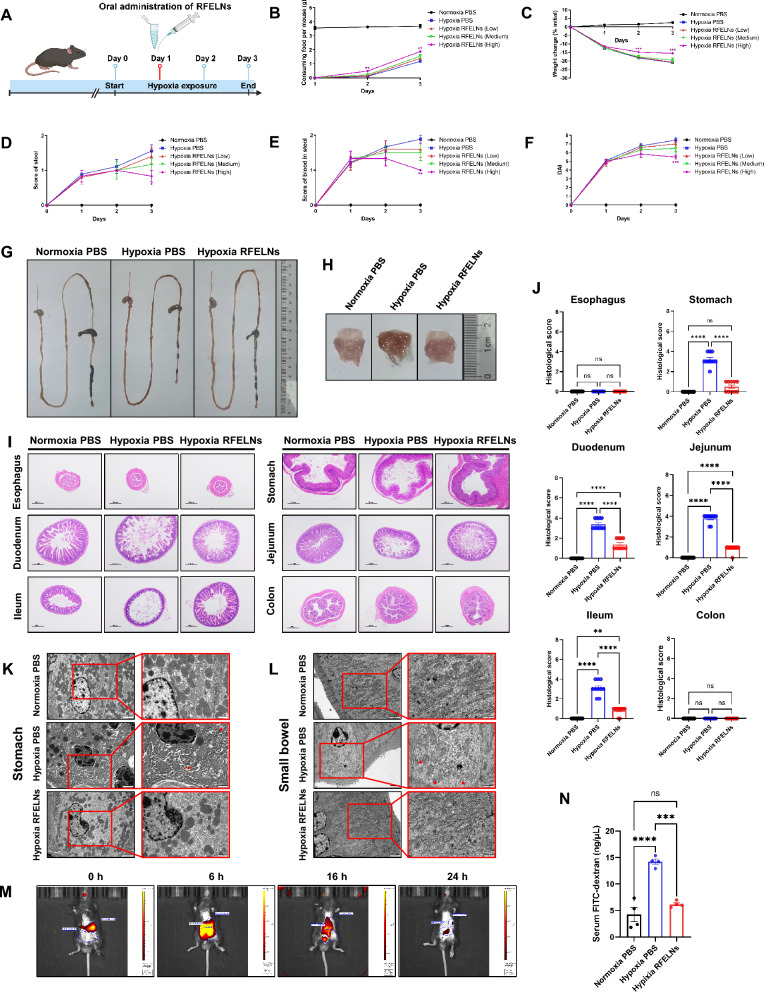
RFELNs alleviated hypoxia-induced gastric and small intestinal mucosal injury in mice. Mice were placed in a ventilated hypoxic chamber system to simulate an altitude of 5500 m above sea level for 3 days (n = 10 per group). PBS or RFELNs (5 × 10^4^ particles, 1 × 10^5^ particles, or 5 × 10^5^ particles) were orally administered on day 1 under hypoxia. Mice were euthanized on day 3. **A** Flowchart of mouse treatments. **B**–**F** Food consumption per mouse, weight change, stool score, stool blood score, and disease activity index were recorded daily. Representative images of the entire gastrointestinal tract (**G**) and stomach (**H**) of mice from each group are shown. **I**, **J** Representative hematoxylin and eosin (H&E)-stained esophagus, stomach, duodenum, jejunum, ileum, and colon tissues (40 × , bar = 500 μm) and histopathological scores. Mitochondrial ultrastructural images of representative cells from gastric (**K**) and small intestinal (**L**) mucosa observed by transmission electron microscopy (TEM) (scale bar, overview 2 μm, inset 1 μm). Mitochondria in the gastric and small intestinal mucosa in the hypoxic PBS group were labeled with red arrows. **M** Biodistribution of DiR-labeled RFELNs in mice after oral administration for 0, 6, 16, or 24 h visualized by in vivo imaging. **N** Assessment of gastrointestinal permeability according to the serum levels of FITC 4 h after FITC–dextran gavage. The RFELNs indicate 5 × 10^5^ particles of RFELNs in G–N. Data indicate the mean ± standard error of the mean (SEM). Green or purple asterisks represent significant differences between RFELNs-treated group (1 × 10^5^ particles or 5 × 10^5^ particles) and PBS control group under hypoxia. *P < 0.05, **P < 0.01, ***P < 0.001, ****P < 0.0001

Mice were orally administered PBS or RFELNs to investigate the therapeutic effect of RFELNs on hypoxia-induced gastric and small intestinal mucosal injury (Fig. 2A). Hypoxia caused bloody stool, loose stool, and weight loss in the mice, and high-dose RFELNs (5 × 10^5^ particles) treatment improved these symptoms (Fig. 2B–F). Compared with those in the normoxia PBS group, the entire gastrointestinal tract and gastric mucosa of the mice in the hypoxia PBS group showed a congested and reddish appearance (Fig. 2G, H). Moreover, the histological analysis indicated significant gastric and small intestinal mucosal barrier disruption, including loss, shedding, and death of gastrointestinal mucosal cells and small villi, shallower crypts, and thinner mucosa of the small bowel induced by hypoxia, while RFELNs administration ameliorated this mucosal damage (Fig. 2I–J, Figure S1). Shrunken mitochondria with increased membrane density and loss of mitochondrial cristae in gastric and small intestinal mucosal cells from hypoxia-exposed mice were observed, a morphologic feature of ferroptosis that can be attenuated by RFELNs administration. (Fig. 2K, L). In addition, murine intestinal permeability was evaluated by oral administration of FITC-dextran. RFELNs administration significantly decreased serum FITC levels, suggesting that RFELNs ameliorated hypoxia-induced gastrointestinal mucosal barrier disruption (Fig. 2N). These results suggested that hypoxia-induced gastric and small intestinal mucosal injury in mice could be significantly alleviated by RFELNs administration. Notably, H&E staining combined with TEM revealed this injury to be closely related to ferroptosis. The accumulation of oral RFELNs in the gastrointestinal tract is essential for exerting therapeutic effects on gastric and small intestinal mucosal injury. Therefore, diR-labeled RFELNs were orally administered and visualized by an in vivo imaging system, revealing an intense fluorescent signal in the whole gastrointestinal tract at 6 h that gradually waned after 16 h (Fig. 2M).

### RFELNs restored the cytokines downregulated by hypoxia while decreasing HIF-1α and peroxide levels

**Fig. 3:**
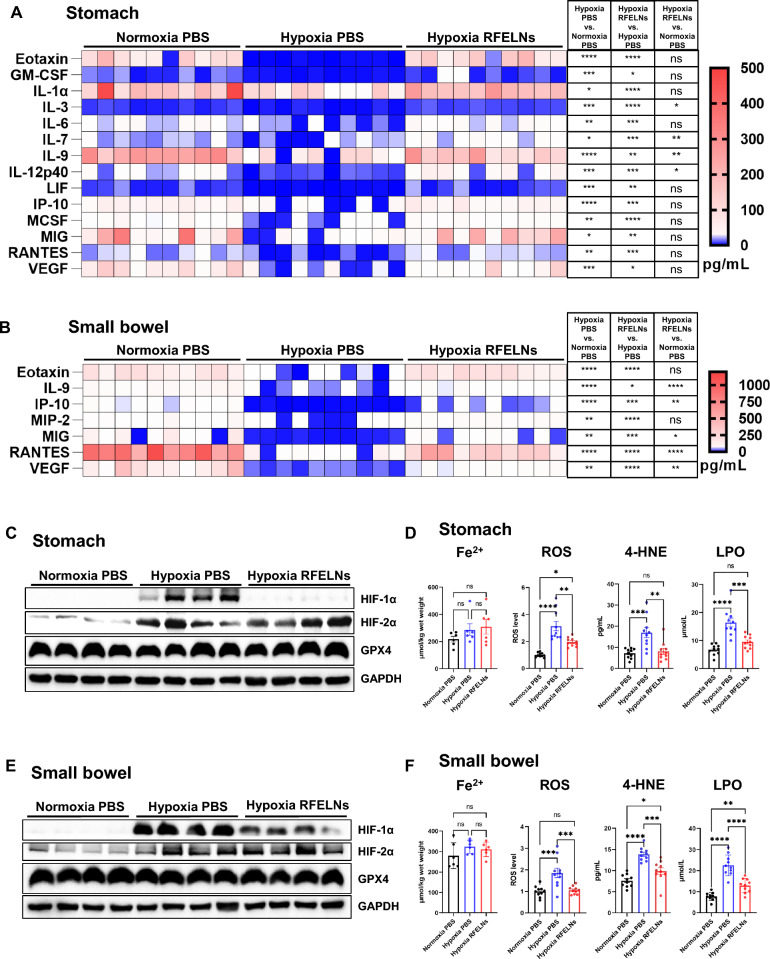
RFELNs restored the decrease in cytokine levels induced by hypoxia and decreased HIF-1α expression and peroxide levels. Mice were exposed to hypoxia at an altitude equivalent to 5500 m above sea level for 3 days (n = 10 per group). PBS or RFELNs (5 × 10^5^ particles) were orally administered on day 1 under hypoxia. The mice were euthanized on day 3. **A** Cytokines in the gastric mucosa. **B** Cytokines in the small intestinal mucosa. **C** The protein expression in the gastric mucosa measured by western blot. **D** The levels of Fe^2+^ and peroxides in the gastric mucosa. **E** Western blot analysis of the protein expression in the small intestinal mucosa. **F** The levels of Fe^2+^ and peroxides in the small intestinal mucosa. The four lanes in each column of the western blot are samples from 4 individual mice; at least 8 mice in each group were included. The data are expressed as the mean ± standard error of the mean (SEM). *P < 0.05, **P < 0.01, ***P < 0.001, ****P < 0.0001

Multiplex ELISA revealed that RFELNs administration restored the levels of eotaxin, GM-CSF, IL-1α, IL-3, IL-6, IL-7, IL-9, IL-12p40, LIF, IP-10, MSCF, MIG, RANTES, and VEGF in the gastric mucosa and eotaxin, IL-9, IP-10, MIP-2, MIG, RANTES, and VEGF in the small intestinal mucosa, which were downregulated by hypoxia (Fig. 3A, B). The gastric and small intestinal mucosal injury caused by hypoxia might be related to ferroptosis (Fig. 2L, M); thus, we measured the levels of HIF-1α, HIF-2α, glutathione peroxidase 4 (GPX4), and peroxides. RFELNs treatment decreased the levels of HIF-1α, ROS, 4-HNE, and LPO, which are activated by hypoxia, in the gastric and small intestinal mucosa without affecting the protein expression of HIF-2α and GPX4 (Fig. 3C–F).

### RFELNs partially improved hypoxia-induced microbial and metabolic disorders of the stomach and small intestine

**Fig. 4:**
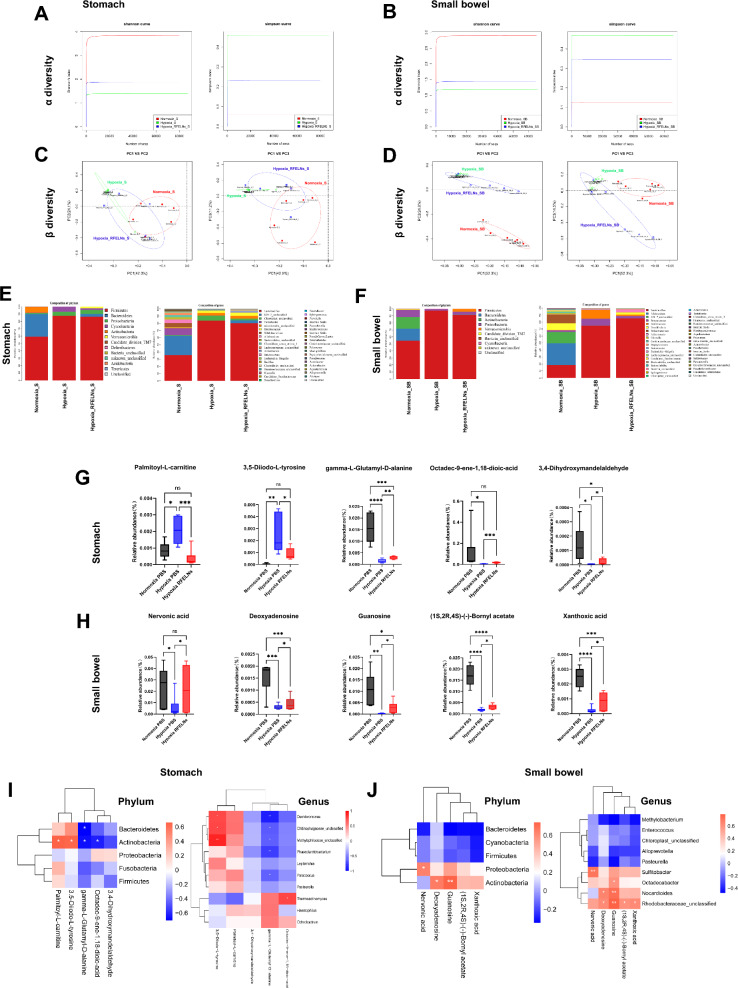
RFELNs partially improved hypoxia-induced microbial and metabolic disorders of the stomach and small bowel. Mice were subjected to hypoxia equivalent to an altitude of 5500 m above sea level for 3 days (n = 6 per group). The mice were orally administered PBS or RFELNs (5 × 10^5^ particles) on day 1 under hypoxia and euthanized on day 3. The microbiota α diversity of gastric (**A**) and small intestinal (**B**) contents. The microbiota β diversity of stomach (**C**) and small intestinal (**D**) contents. The relative abundance of the microbiota community composition of the gastric (**E**) and small intestinal (**F**) contents at the phylum and genus levels. Changes in metabolites in the gastric (**G**) and small intestinal (**H**) contents. Integrated analysis of the microbiota and metabolites of gastric (**I**) and small intestinal (**J**) contents. S = Stomach in A, C, E, *SB* Small bowel in B, D, F. Data are shown as the mean ± standard error of the mean (SEM). *P < 0.05, **P < 0.01, ***P < 0.001, ****P < 0.0001

Considering the strong association between the microbiota metabolites and the gastrointestinal mucosal barrier, we analyzed the microbiota and metabolites of gastric and small intestinal contents from mice treated as indicated above. RFELNs ameliorated the hypoxia-induced reductions in the α-diversity of the gastric and small intestinal microbiota, and the β-diversity of the gastric microbiota without significantly improving the β-diversity of the small intestinal microbiota (Fig. 4A–D). Then, the differential community composition was determined in terms of phylum and genus (Fig. 4E, F), and the microbiota significantly associated with differentially abundant metabolites were further quantified. In the stomach, *Actinobacteria* and *Bacteroidetes* were notably decreased at the phylum level under hypoxia. At the genus level, the abundances of *Chitinophagaceae_unclassified, Dechloromonas, Methylophilaceae_unclassified, Paracoccus, and Phascolarctobacterium* increased, and the abundance of Thermoactinomyces decreased under hypoxia. In the small bowel, the abundances of *Actinobacteria* and *Proteobacteria* decreased at the phylum level, and the abundances of *Nocardioides, Octadecabacter, Rhodobacteraceae_unclassified, and Sulfitobacter* decreased at the genus level under hypoxia. RFELNs treatment partially reversed the abovementioned microbiota disorders (Figure S2A, B). The differentially abundant metabolites from the stomach and small intestine were further analyzed, revealing that RFELNs partially restored dysregulated palmitoyl-L-carnitine, 3,5-diiodo-L-tyrosine, γ-L-glutamyl-D-alanine, octadec-9-ene-1,18-dioic-acid, and 3,4-dihydroxymandelaldehyde in the stomach exposed to hypoxia (Fig. 4G) and partially restored abnormal nervonic acid, deoxyadenosine, guanosine, (1S,2R,4S)-(-)-bornyl acetate, and xanthoxic acid in the small intestine under hypoxia (Fig. 4H). Correlation analyses of differentially abundant metabolites and differential microbiota in the stomach and small intestine were performed. Combined microbial and metabolomic analysis revealed that at the phylum level, the abundance of *Bacteroidetes* was significantly negatively correlated with the abundance of γ-L-glutamyl-D-alanine. *Actinobacteria* was negatively correlated with γ-L-glutamyl-D-alanine and octadec-9-ene-1,18-dioic-acid and significantly positively correlated with palmitoyl-L-carnitine and 3,5-diiodo-L-tyrosine in the stomach. In addition, *Proteobacteria* and *Actinobacteria* were significantly positively correlated with neuronic acid, deoxyadenosine, and guanosine in the small intestine. At the genus level, in the stomach, 3,5-diiodo-L-tyrosine was significantly positively correlated with *Dechloromonas, Chitinophagaceae_unclassified*, and *Methylophilaceae_unclassified*. γ − L − Glutamyl − D − alanine was significantly negatively correlated with *Dechloromonas*, *Chitinophagaceae_unclassified*, *Methylophilaceae_unclassified, Phascolarctobacterium*, and *Paracoccus*. Octadec-9-ene-1,18-dioic acid was significantly positively correlated with *Thermoactinomyces*. In the small intestine, Nervonic acid and deoxyadenosine were significantly positively correlated with *Sulfitobacter, Rhodobacteraceae_unclassified,* and *Nocardioides*. Guanosine was significantly positively correlated with *Octadecabacter, Nocardioides, and Rhodobacteraceae_unclassified.* (1S,2R,4S)-(-)-Bornyl acetate and xanthoxic acid were significantly positively correlated with *Rhodobacteraceae_unclassified* (Fig. 4I, J).

### RFELNs suppressed hypoxia-induced ferroptosis in NGEC and HIEC

**Fig. 5:**
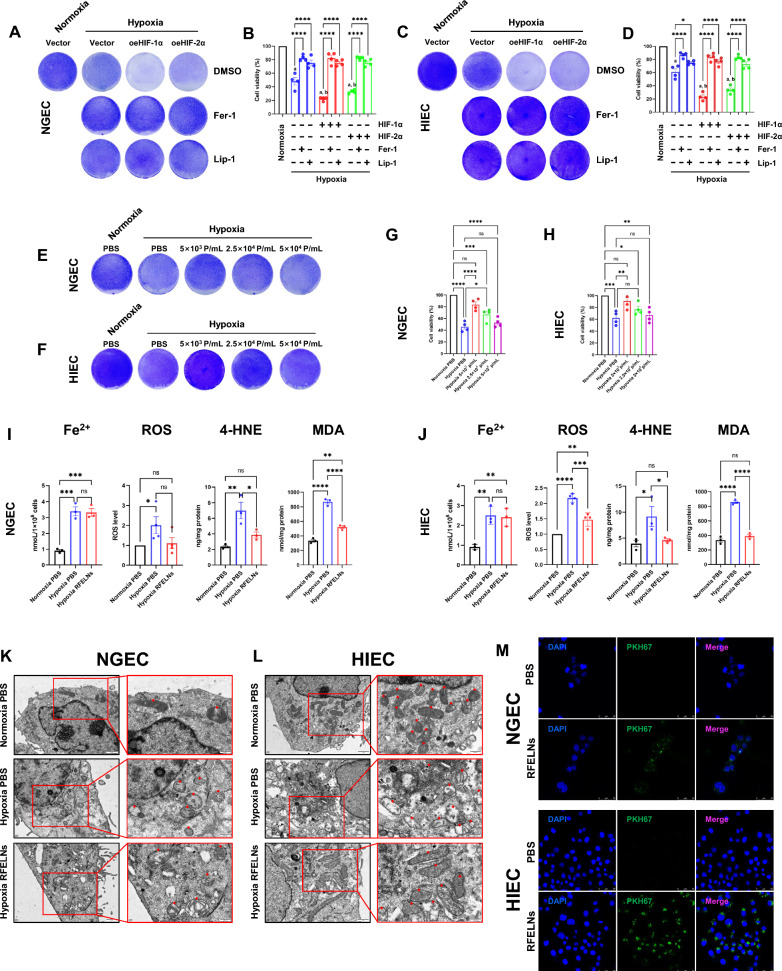
RFELNs suppressed hypoxia-induced ferroptosis in NGEC and HIEC. NGEC and HIEC were subjected to hypoxia (1% O_2_). For transfection experiments, NGEC and HIEC were transiently transfected with empty vector, HIF-1α, or HIF-2α plasmid. 24 h after transfection under normoxia, the cells were incubated under hypoxia for 24 h, and then the cells were treated with dimethyl sulfoxide (DMSO), ferrostatin-1 (Fer-1, 5 μM), or liproxstatin-1 (Lip-1, 1 μM) under hypoxia for another 24 h. NGEC and HIEC were harvested for crystal violet staining (**A**, **C**) and cell viability assessments (**B**, **D**). For RFELNs treatment experiments, NGEC and HIEC were subjected to different concentrations of RFELNs or PBS after 24 h of hypoxia. Then, NGEC and HIEC were collected for crystal violet staining (**E**, **F**) and cell viability assessments (**G**, **H**) after further exposure to hypoxia for 24 h. Fe^2+^, ROS, 4-HNE, and MDA levels in NGEC (**I**) and HIEC (**J**) were measured. Mitochondrial ultrastructural images of the representative cells from NGEC (**K**) and HIEC (**L**) were observed by transmission electron microscopy (TEM) (scale bar, overview 1 μm, inset 500 nm). The mitochondria of NGEC and HIEC were labeled with red arrows. The distribution of PKH67-labeled RFELNs after incubation with NGEC and HIEC for 24 h was visualized using a confocal microscope (PKH67, green; DAPI, blue; bar = 50 μm). RFELNs (5 × 10^3^ particles/mL) were applied to M. P/mL = Particles/mL. The data are presented as the mean ± standard errors of the mean (SEMs). Hypoxia + vector, hypoxia + HIF-1α, or hypoxia + HIF-2α group vs. Normoxia control, ^a^ *P* < 0.0001 in B, D. Hypoxia + HIF-1α or hypoxia + HIF-2α group vs. hypoxia + vector group, ^b^ *P* < 0.05 at least in B, D. *P < 0.05, **P < 0.01, ***P < 0.001, and ****P < 0.0001. A representative of at least three independent experiments is shown

Animal experiments suggested that gastric and small intestinal mucosal damage may be related to HIF-1α- and HIF-2α-mediated ferroptosis; thus, hypoxic cells were transfected with HIF-1α or HIF-2α plasmids and treated with ferroptosis inhibitors to determine whether HIF-1α and HIF-2α could lead to ferroptosis in the stomach and small intestine. The results of crystal violet staining and CCK-8 assays showed that overexpression of HIF-1α or HIF-2α exacerbated hypoxia-induced cell death in NGEC and HIEC (Fig. 5A–D). The ferroptosis inhibitors fer-1 and lip-1 significantly reversed the cell death induced by overexpression of HIF-1α or HIF-2α under hypoxia (Fig. 5A–D), indicating that the mode of cell death induced by hypoxia was predominantly ferroptosis. Moreover, RFELNs significantly reversed hypoxia-induced cell death in NGEC and HIEC (Fig. 5E–H). Subsequently, cellular peroxidation levels were examined, and the results showed that hypoxia exacerbated the levels of ROS, 4-HNE, and MDA in cells. By contrast, after RFELNs treatment, the levels of these ferroptosis peroxidation markers were obviously decreased (Fig. 5I, J), consistent with our mitochondrial electron microscopy results (Fig. 5K, L). In addition, confocal microscopy revealed that the SFELNs were engulfed by NGEC and HIEC (Fig. 5M).

### RFELNs inhibited HIF-1α- and HIF-2α-mediated NOX4 and ALOX5, alleviating ferroptosis induced by hypoxia in NEGC and HIEC

**Fig. 6:**
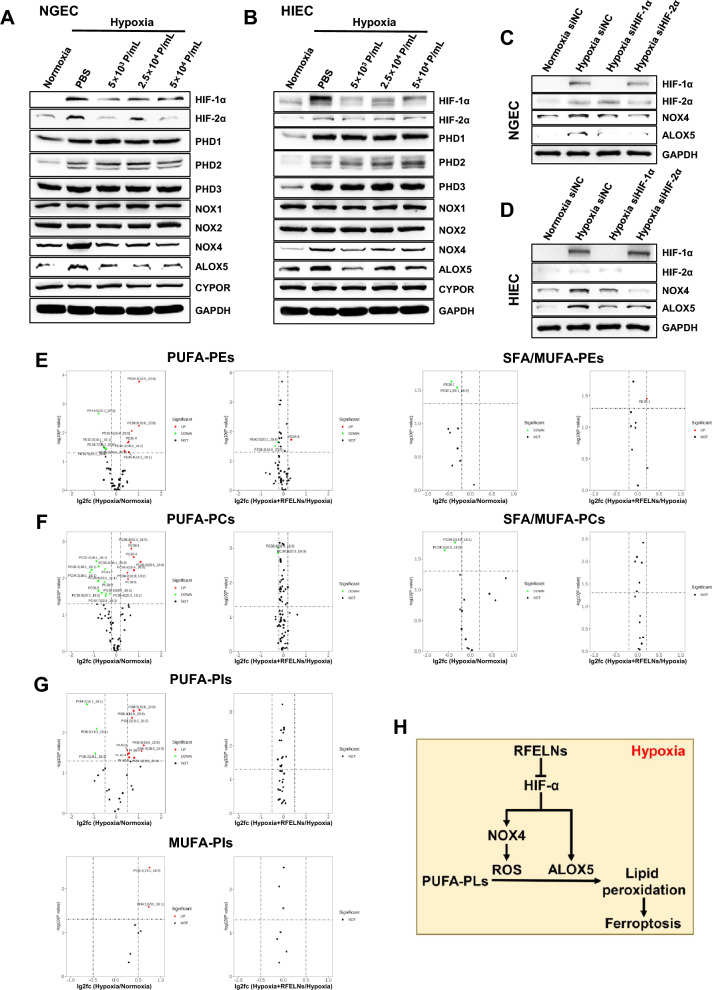
RFELNs inhibited HIF-1α- and HIF-2α-mediated NOX4 and ALOX5, alleviating ferroptosis caused by hypoxia. For the RFELNs treatment experiments, NGEC and HIEC were incubated for 24 h under hypoxia (1% O_2_) and subsequently treated with different RFELNs or PBS for 24 h under hypoxia. For knockdown experiments, NGEC and HIEC were transiently transfected with siNC, siHIF-1α, or siHIF-2α. 24 h after transfection under normoxia, the cells were incubated under hypoxic conditions for 48 h. The protein expression levels in NGEC (**A**, **C**) and HIEC (**B**, **D**) were measured by western blot. **E** Volcano plots showing the changes in PUFA-PE and SFA/MUFA-PE between the indicated groups (n = 3). **F** Volcano plots showing the changes in PUFA-PCs and SFA/MUFA-PCs between the indicated groups (n = 3). **G** Volcano plots highlighting the changes in PUFA-PIs and MUFA-PIs between the indicated groups (n = 3). **H** Diagram of the mechanism by which RFELNs inhibit hypoxia-induced ferroptosis in gastric and small intestinal mucosal cells. RFELNs (5 × 10^3^ particles/mL) were applied for lipidomic analysis. P/mL = Particles/mL in A & B. Data are represented as the mean ± standard error of the mean (SEM). A representative of at least three independent experiments is shown in A–D. *PUFA* polyunsaturated fatty acids, *SFA* saturated fatty acids, *MUFA* monounsaturated fatty acids, *PE* phosphatidylethanolamine, *PC* phosphatidylcholine, *PI* phosphatidylinositol

To determine how RFELNs inhibited hypoxia-mediated ferroptosis in NGEC and HIEC, HIF-α, PHDs, enzymes involved in PUFA peroxidation and ROS production, and lipid metabolism were analyzed. The inhibitory effects of RFELNs on HIF-1α and HIF-2α expression were not altered by PHDs (Fig. 6A, B). Hypoxia-induced NOX4 and ALOX5 expression was significantly downregulated by RFELNs, whereas NOX1, NOX2, and CYPOR exhibited no obvious changes among the different groups. Notably, the band of ALOX12 and ALOX15 was not detected in NGEC or HIEC. Therefore, HIF-1α or HIF-2α was knocked down in NGEC and HIEC to determine whether HIF-1α or HIF-2α regulates ALOX5 and NOX4. ALOX5 and NOX4 expression levels were reduced after inhibiting HIF-1α or HIF-2α (Fig. 6C, D). Ferroptosis is characterized by peroxidized membrane PLs, particularly PUFA-containing PLs. Thus, we detected phosphatidylethanolamines (PEs), phosphatidylcholines (PCs), and phosphatidylinositols (PIs) containing PUFAs and saturated fatty acids (SFAs)/monounsaturated fatty acids (MUFAs) in HIEC subjected to normoxia, hypoxia, or hypoxia plus RFELNs. Considering that PUFA-PLs sensitize cells to ferroptosis while MUFA-PLs inhibit ferroptosis by reducing the abundance of PUFA-PLs, PIs and PEs were considered the main PLs involved in ferroptosis caused by hypoxia in HIEC (Fig. 6E–G). However, RFELNs failed to exert significant effects on hypoxia-induced changes in PLs.

## Discussion

An increasing number of recent studies have been conducted on PELNs since most are edible and have profound potential for use in drug delivery systems with minimal cytotoxicity and side effects [46]. Robinia pseudoacacia L. flowers are commonly consumed as medical and edible plants. In this study, RFELNs were extracted from Robinia pseudoacacia L. flowers. Oral RFELNs treatment effectively alleviated hypoxia-induced ferroptosis in the gastric and small intestinal mucosal cells and partially alleviated hypoxia-induced microbial and metabolic disorders. In addition, RFELNs exhibited specific targeting to the gastrointestinal tract.

Reduced food intake, weight loss, diarrhea, and fecal occult blood, which have been reported to be common symptoms of digestive disorders caused by high-altitude hypoxic environments [1–3], were relatively objective indexes to assess hypoxia-induced gastric and small intestinal mucosal injury in this study. Anorexia may be related to the downregulation of neuropeptide Y and galanin [47], and impaired gastric and small intestinal mucosa. A damaged gastrointestinal mucosal barrier, which impairs nutrient absorption, along with anorexia, cause weight loss [48]. Damage to the small intestinal mucosa could lead to digestive and absorptive dysfunction, electrolyte imbalance, and then diarrhea [49]. Gastric and small intestinal mucosal injury can result in fecal occult blood.

Cellular metabolism in hypoxic environments is dependent mainly on the regulation of HIFs. PHDs are inhibitors of HIFs that induce the polyubiquitination and degradation of HIF-α, and the inactivation of PHDs promotes the stabilization and expression of HIF-α in hypoxic environments [50]. Increased HIF-α levels are a double-edged sword. HIF-α can enhance cellular adaptation to hypoxic environments under acute hypoxic conditions [6]; however, if hypoxia persists, high HIF-α expression will lead to cell death [7]. In this study, hypoxia led to gastric and small intestinal mucosal ferroptosis and increased HIF-1α and HIF-2α expression, consistent with the results of other studies [51, 52]. In vivo experiments demonstrated that the oral administration of RFELNs alleviated hypoxia-induced gastric and small intestinal mucosal injury and restored the downregulated cytokine levels induced by hypoxia while decreasing the levels of HIF-1α and peroxides. Our histological analysis and peroxide analysis findings indicated that the decrease in gastric and small intestinal cytokine levels might result from ferroptosis of immune cells in the lamina propria, which was suppressed by RFELNs treatment. In hypoxic environments in vitro, the overexpression of HIF-1α or HIF-2α exacerbated hypoxia-induced cell death, and this effect was significantly reversed by ferroptosis inhibitors. RFELNs restored hypoxia-induced increases in ROS and peroxide levels, indicating that the mode of cell death induced by hypoxia was predominantly HIF-1α- and HIF-2α-mediated ferroptosis, which could be inhibited by RFELNs. Western blot revealed a significant decrease in NOX4 and ALOX5 expression, accompanied by a decrease in HIF-1α and HIF-2α expression after RFELNs treatment. The inhibitory effects of RFELNs on the expression of HIF-1α and HIF-2α were not reduced by PHDs. Further inhibition of HIF-1α or HIF-2α expression with siRNA also decreased NOX4 and ALOX5 expression, and NOX4 and ALOX5 participate in driving ROS production and lipid peroxidation, respectively [20–22], suggesting that RFELNs inhibit ferroptosis by suppressing HIF-1α- and HIF-2α-mediated lipid peroxidation. However, the effect of RFELNs on PUFA-PLs in HIEC was not obvious for the following two reasons: (1) alterations in PUFA-PLs under hypoxia are not mediated by HIF-α, and (2) certain RFELN components affect HIF-α-mediated PUFA-PLs. RFELNs downregulated HIF-1α expression but not HIF-2α expression in the gastric and small intestinal mucosa of mice. However, RFELNs inhibited both HIF-1α and HIF-2α in the human cell lines NGEC and HIEC. These differences are potentially species-dependent. It should be mentioned that, RFELNs couldn’t downregulate Fe^2+^ to inhibit ferroptosis.

Oral RFELNs treatment alleviated hypoxia-induced microbial and metabolic disorders to a certain extent. 16S rRNA sequencing indicated that RFELNs restored hypoxia-induced reductions in the α and β diversity of the gastric microbiota and the α diversity of the small intestinal microbiota but did not significantly restore the reduction in the β diversity of the small intestinal microbiota. Metabolite analyses revealed that RFELNs treatment restored hypoxia-induced increases in palmitoyl-L-carnitine and 3,5-diiodo-l-tyrosine and decreases in 3,4-dihydroxymandelaldehyde, gamma-L-glutamyl-D-alanine, and octadec-9-ene-1,18-dioic-acid in the stomach. High concentrations of palmitoyl-L-carnitine affect mitochondrial function and increase ROS [53]. 3,5-Diiodo-l-tyrosine, an intermediate of 3,5,3′,5′-tetraiodo-l-thyronine, can inhibit tyrosine hydroxylase (TH) [54], while hypoxia stimulates the expression of the TH gene [55]. 3,4-Dihydroxymandelaldehyde is negatively associated with anxiety and depression in patients with irritable bowel syndrome, and its reduction may reflect an imbalance in the brain–gut axis [56]. In addition, RFELNs elevated the hypoxia-induced decreases in deoxyadenosine, guanosine, (1S,2R,4S)-(-)-Bornyl acetate, xanthoxic acid, and nervonic acid in the small intestine. Deoxyadenine is a nucleoside composed of deoxyadenine and deoxyribose. When DNA is damaged due to hypoxia, cells initiate DNA repair mechanisms, which potentially decreases deoxyadenosine [57]. Nervonic acid and guanosine can inhibit oxidative stress by downregulating ROS production [58, 59]. In the stomach, RFELNs restored hypoxia-induced increase in Dechloromonas, Methylophilaceae_unclassified, Paracoccus, and Phascolarctobacterium, and decrease in Thermoactinomyces, suggesting that Dechloromonas, Methylophilaceae_unclassified, Paracoccus, and Phascolarctobacterium might be detrimental while Thermoactinomyces might exert beneficial effects under hypoxia. In the small bowel, RFELNs restored hypoxia-induced decrease in Rhodobacteraceae_unclassified, indicating that Rhodobacteraceae_unclassified might be beneficial under hypoxia. Combined microbial and metabolomic analysis showed that, Thermoactinomyces was positively correlated with octadec-9-ene-1,18-dioic-acid, 3,5-diiodo-L-tyrosine was positively correlated with Dechloromonas and Methylophilaceae_unclassified, γ-l-glutamyl-d-alanine was negatively correlated with Dechloromonas, Methylophilaceae_unclassified, Paracoccus, and Phascolarctobacterium, in the stomach. Rhodobacteraceae_unclassified was positively correlated with deoxyadenosine, guanosine, (1S,2R,4S)-(-)-bornyl acetate, and xanthoxic acid, in the small intestine. These results suggested that octadec-9-ene-1,18-dioic-acid, γ-l-glutamyl-d-alanine, deoxyadenosine, guanosine, (1S,2R,4S)-(-)-bornyl acetate, and xanthoxic acid might offer benefits while 3,5-diiodo-L-tyrosine might be deleterious under hypoxia.

Why was significant damage observed in the gastric and small intestinal mucosa but not the esophagus or colon? Although few studies have investigated the partial pressure of oxygen (pO_2_) in the esophageal mucosa, it has been reported that the pO_2_ in the colonic mucosa is significantly lower than that in the gastric and small intestinal mucosa [60]. Therefore, we speculate that the esophageal and colonic mucosa have a better tolerance to hypoxia. We intend to measure pO_2_ in the esophageal mucosa to validate our hypothesis in subsequent experiments.

HIFs play an important role in regulating basic cellular metabolism processes, such as tumorigenesis, vascular proliferation, inflammation regulation, and anaerobic metabolism [61, 62]. Inhibiting HIFs suppresses tumor growth, vascularization, metabolic reprogramming, invasion, metastasis, and resistance to radiation therapy and chemotherapy in cancer cells [63]. Preclinical studies have shown that HIFs inhibition can treat inflammatory diseases such as sepsis, inflammatory bowel disease, and rheumatoid arthritis [64–67]. Our research revealed that RFELNs inhibited HIF-1α in vivo and both HIF-1α and HIF-2α in vitro, providing a broad perspective relevant to research on diseases associated with elevated HIFs.

This study has several limitations. First, we did not identify the specific effective components of RFELNs. Combining microRNA sequencing with RNAhybrid and TargetScan databases, we found that microRNAs in RFELNs contain binding sites for both HIFs and PHDs (data not shown), making it difficult for RFELNs to reduce HIFs through microRNAs. Therefore, the specific effective components of RFELNs are likely metabolites or proteins, which we will investigate in future studies.

In summary, we successfully isolated and identified RFELNs from Robinia pseudoacacia L. flowers and found that RFELNs treatment effectively alleviated hypoxia-induced ferroptosis in gastric and small intestinal mucosal cells by suppressing HIF-1α- and HIF-2α-mediated lipid peroxidation. In addition, RFELNs partially alleviated hypoxia-induced microbial and metabolic disorders. These results suggest that oral RFELNs demonstrate potential as therapeutics for gastric and intestinal mucosal injury under hypoxia.

### Supplementary Information

Additional file 1.

## Data Availability

All data generated or analyzed during this study are included in this published article and its supplementary information files.

## Abbreviations

HIFs: Hypoxia-inducible factors
PUFA: Polyunsaturated fatty acids
PLs: Phospholipids
ROS: Reactive oxygen species
PHDs: Prolyl hydroxylases
NOX: Nicotinamide adenine dinucleotide phosphate oxidase
ALOX: Arachidonate lipoxygenase
POR, also known as CYPOR: P450 oxidoreductase
PELNs: Plant exosome-like nanoparticles
RFELNs: Robinia pseudoacacia L. flowers exosome-like nanoparticles
PBS: Phosphate-buffered saline
TEM: Transmission electron microscopy
H & E: Hematoxylin and eosin
DAI: Disease activity index
NGEC: Human normal gastric epithelial cell line
HIEC: Human normal small intestinal epithelial cell line
CCK-8: Cell counting kit-8
DMSO: Dimethyl sulfoxide
OTUs: Operational taxonomic units
LC–MS: Liquid chromatography and mass spectrum
DAG: Diacylglycerols
TAG: Triacylglycerols
GPX4: Glutathione peroxidase 4
4-HNE: 4-Hydroxynonenal
LPO: Lipid hydroperoxide
MDA: Malondialdehyde
pO_2_: Partial pressure of oxygen
PC: Phosphatidylcholine
PI: Phosphatidylinositol
PE: Phosphatidylethanolamine
SFA: Saturated fatty acids
MUFA: Monounsaturated fatty acids
