# Supplementary material for: Oral administration of Robinia pseudoacacia L. flower exosome-like nanoparticles attenuates gastric and small intestinal mucosal ferroptosis caused by hypoxia through inhibiting HIF-1α- and HIF-2α-mediated lipid peroxidation
Source: J Nanobiotechnology. 2024 Aug 12;22:479. doi: 10.1186/s12951-024-02663-6 (PMC11321022; doi:10.1186/s12951-024-02663-6)
Supplement: Supplementary file 1 — Additional file 1. [file 12951_2024_2663_MOESM1_ESM.pptx]

## Slide 1
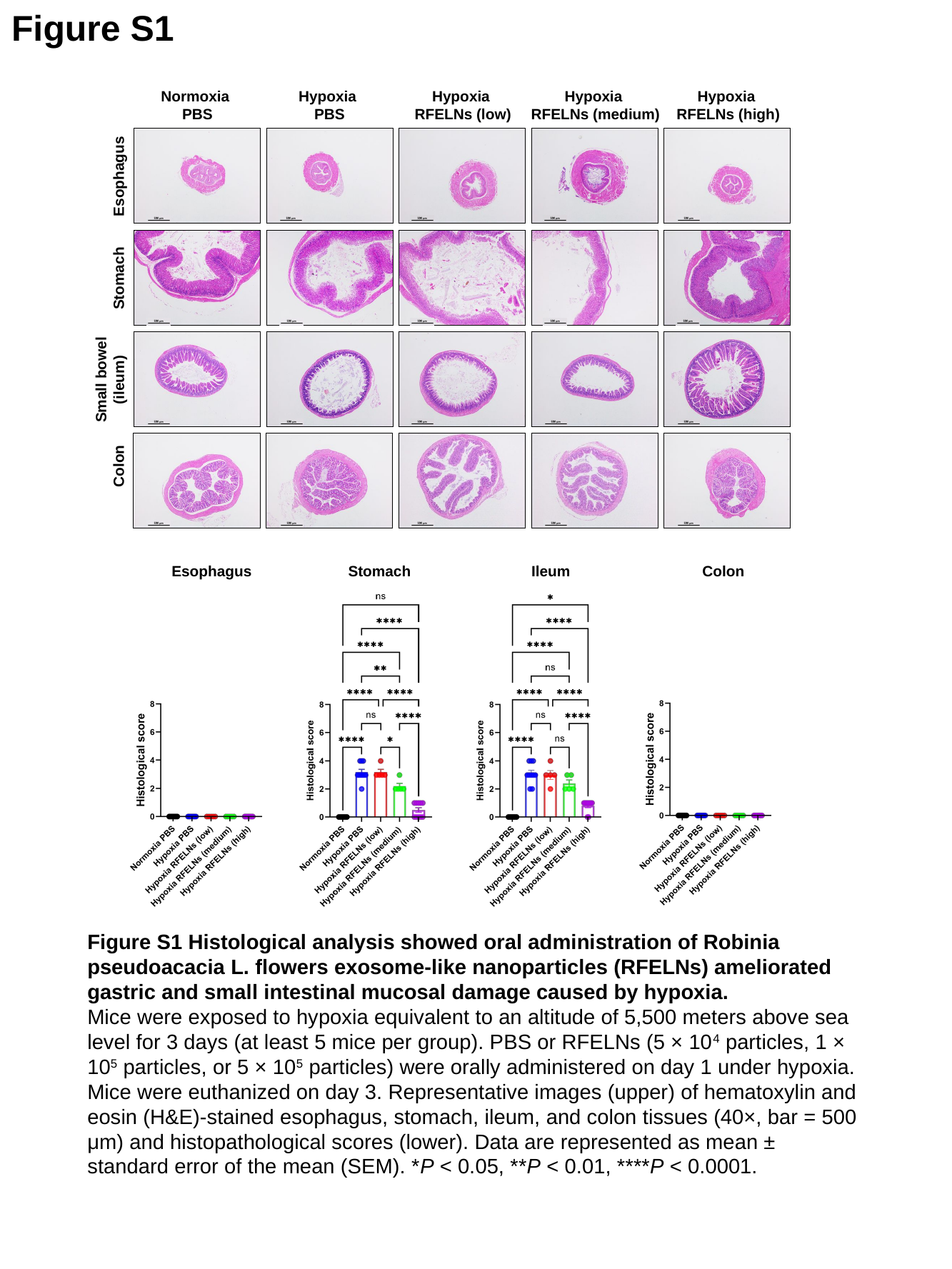

Figure S1
Normoxia
PBS
Hypoxia
PBS
Hypoxia
RFELNs (low)
Hypoxia
RFELNs (medium)
Hypoxia
RFELNs (high)
Esophagus
Stomach
Small bowel
(ileum)
Colon
Esophagus
Stomach
Ileum
Colon
Figure S1 Histological analysis showed oral administration of Robinia pseudoacacia L. flowers exosome-like nanoparticles (RFELNs) ameliorated gastric and small intestinal mucosal damage caused by hypoxia.
Mice were exposed to hypoxia equivalent to an altitude of 5,500 meters above sea level for 3 days (at least 5 mice per group). PBS or RFELNs (5 × 10^4^ particles, 1 × 10^5^ particles, or 5 × 10^5^ particles) were orally administered on day 1 under hypoxia. Mice were euthanized on day 3. Representative images (upper) of hematoxylin and eosin (H&E)-stained esophagus, stomach, ileum, and colon tissues (40×, bar = 500 μm) and histopathological scores (lower). Data are represented as mean ± standard error of the mean (SEM). *P < 0.05, **P < 0.01, ****P < 0.0001.

## Slide 2
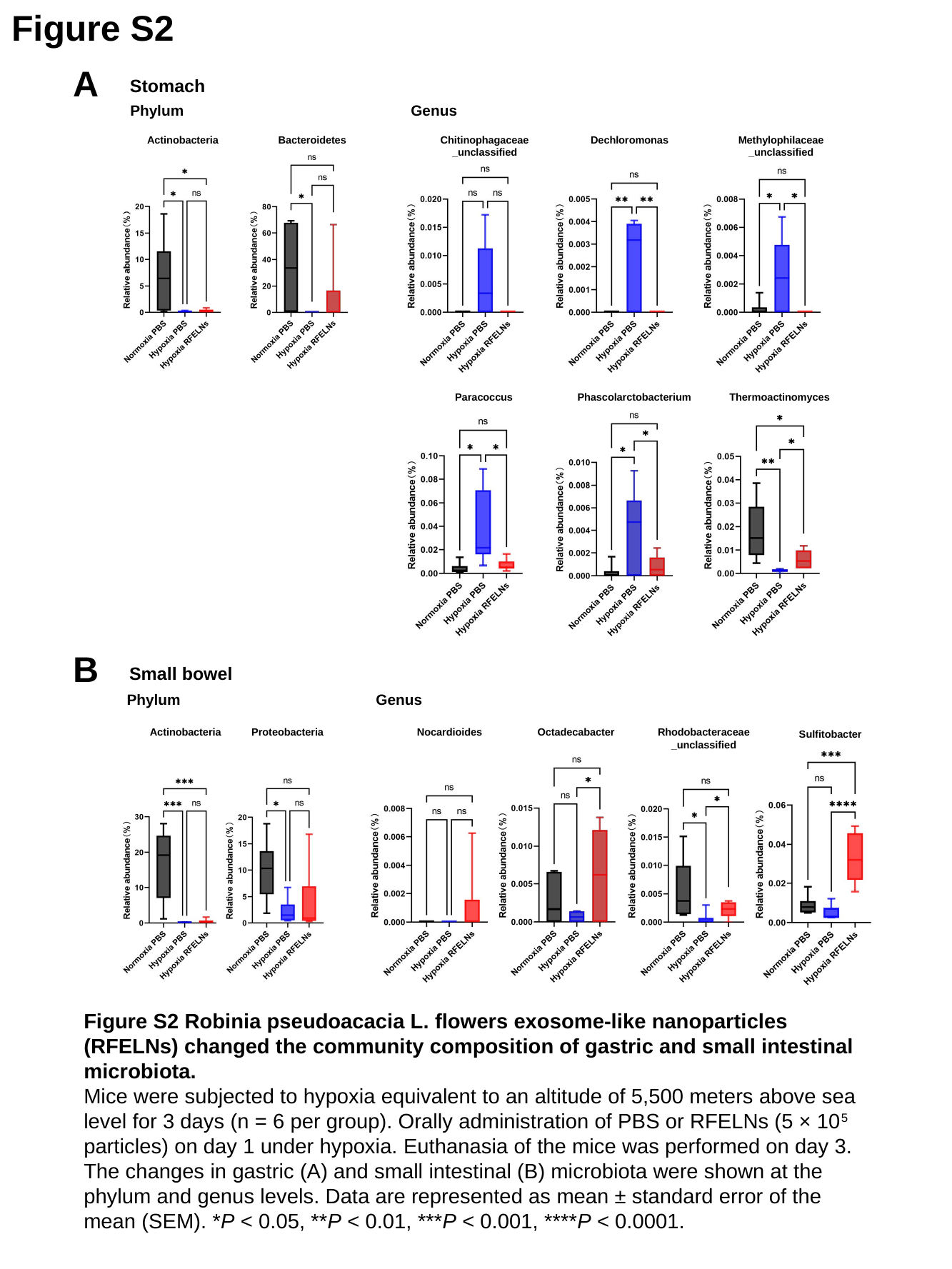

Figure S2
A
Stomach
Phylum
Genus
Actinobacteria
Bacteroidetes
Chitinophagaceae
_unclassified
Dechloromonas
Methylophilaceae
_unclassified
Paracoccus
 Phascolarctobacterium
Thermoactinomyces
B
Small bowel
Phylum
Genus
Actinobacteria
Proteobacteria
Nocardioides
Octadecabacter
Rhodobacteraceae
_unclassified
Sulfitobacter
Figure S2 Robinia pseudoacacia L. flowers exosome-like nanoparticles (RFELNs) changed the community composition of gastric and small intestinal microbiota.
Mice were subjected to hypoxia equivalent to an altitude of 5,500 meters above sea level for 3 days (n = 6 per group). Orally administration of PBS or RFELNs (5 × 10^5^ particles) on day 1 under hypoxia. Euthanasia of the mice was performed on day 3. The changes in gastric (A) and small intestinal (B) microbiota were shown at the phylum and genus levels. Data are represented as mean ± standard error of the mean (SEM). *P < 0.05, **P < 0.01, ***P < 0.001, ****P < 0.0001.

## Slide 3
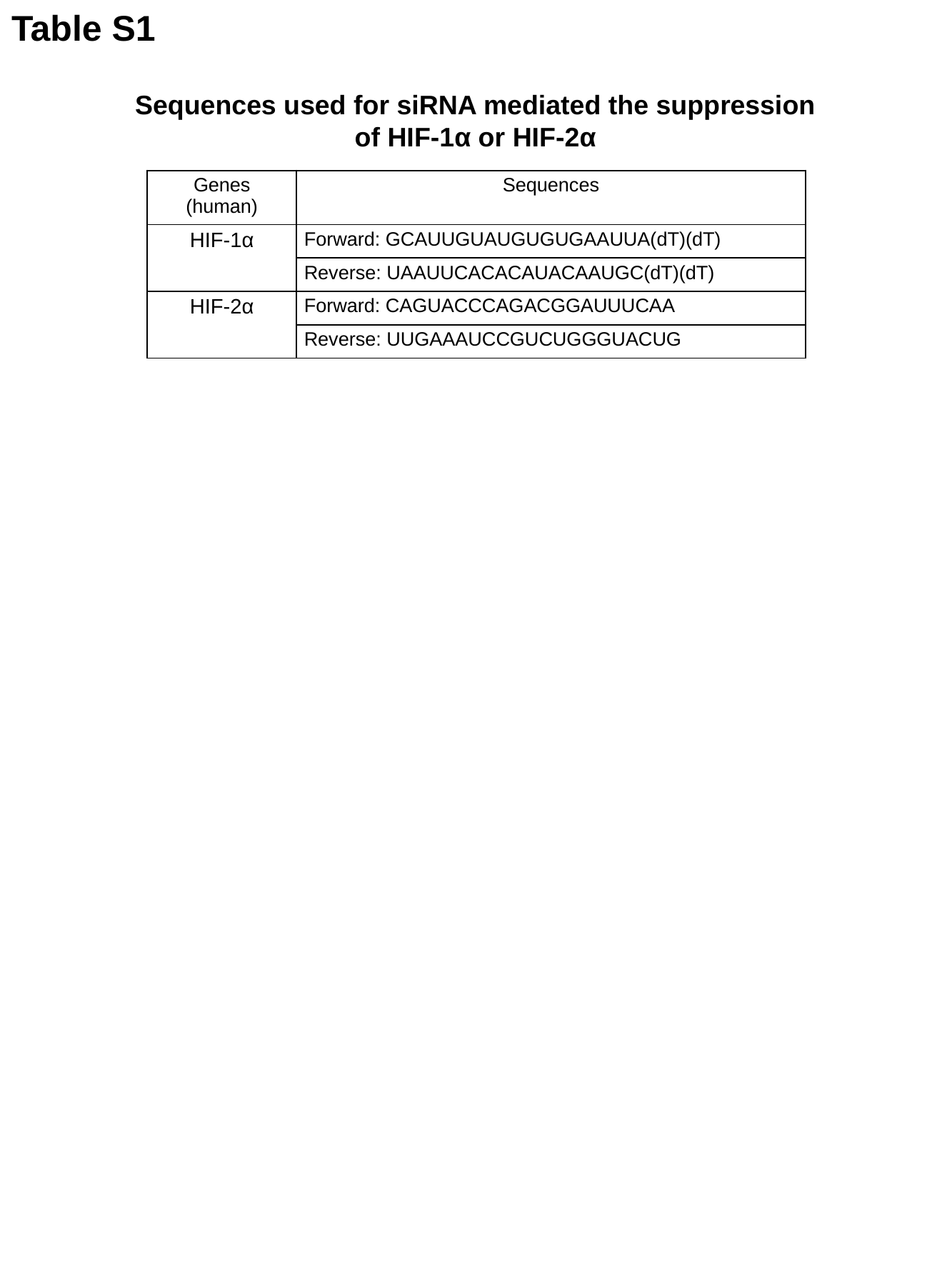

Table S1
Sequences used for siRNA mediated the suppression of HIF-1α or HIF-2α
| Genes (human) | Sequences |
| --- | --- |
| HIF-1α | Forward: GCAUUGUAUGUGUGAAUUA(dT)(dT) |
| | Reverse: UAAUUCACACAUACAAUGC(dT)(dT) |
| HIF-2α | Forward: CAGUACCCAGACGGAUUUCAA |
| | Reverse: UUGAAAUCCGUCUGGGUACUG |
